# Acute effects of a single bout of exercise therapy on knee acoustic emissions in patients with osteoarthritis: a double-blinded, randomized controlled crossover trial

**DOI:** 10.1186/s12891-022-05616-y

**Published:** 2022-07-11

**Authors:** Kristin Kalo, Daniel Niederer, Marco Schmitt, Lutz Vogt

**Affiliations:** 1 Departement of Sports Medicine, Disease Prevention and Rehabilitation, Johannes Gutenberg University Mainz, Albert-Schweitzer-Straße 22, 55128 Mainz, Germany; 2 Department of Sports Medicine and Exercise Physiology, Institute of Occupational, Social and Environmental Medicine, Goethe University Frankfurt, Frankfurt am Main, Germany; 3 Department of Sports Medicine and Exercise Physiology, Goethe University Frankfurt, Frankfurt am Main, Germany

**Keywords:** Vibroarthrography, Knee joint sound, Neuromuscular exercise, Knee osteoarthritis, Activities of daily living

## Abstract

**Background:**

Knee osteoarthritis is associated with higher kinetic friction in the knee joint, hence increased acoustic emissions during motion. Decreases in compressive load and improvements in movement quality might reduce this friction and, thus, sound amplitude. We investigated if an exercise treatment acutely affects knee joint sounds during different activities of daily life.

**Methods:**

Eighteen participants with knee osteoarthritis (aged 51.8 ± 7.3 years; 14 females) were included in this randomized crossover trial. A neuromuscular exercise intervention and a placebo laser needle acupuncture treatment were performed. Before and after both interventions, knee joint sounds were measured during three different activities of daily living (standing up/sitting down, walking, descending stairs) by means of vibroarthrography. The mean amplitude (dB) and the median power frequency (MPF, Hz) were assessed at the medial tibial plateau and the patella. Differences in knee acoustic emissions between placebo and exercise interventions were calculated by analyses of covariance.

**Results:**

Controlled for participant's age, knee demanding activity level and osteoarthritis stage, the conditions significantly differed in their impact on the MPF (mean(± SD) pre-post-differences standing up: placebo: 9.55(± 29.15) Hz/ exercise: 13.01(± 56.06) Hz, F = 4.9, *p* < 0.05) and the amplitude (standing up: placebo:0.75(± 1.43) dB/ exercise: 0.51(± 4.68) dB, F = 5.0, *p* < 0.05; sitting down: placebo: 0.07(± 1.21) dB/ exercise: -0.16(± .36) dB, F = 4.7, *p* < 0.05) at the tibia. There were no differences in the MPF and amplitude during walking and descending stairs (*p* > 0.05). At the patella, we found significant differences in the MPF during walking (placebo 0.08(± 1.42) Hz/ exercise: 15.76(± 64.25) Hz, F = 4.8, *p* < .05) and in the amplitude during descending stairs (placebo: 0.02 (± 2.72) dB/ exercise: -0.73(± 2.84) dB, F = 4.9, *p* < 0.05). There were no differences in standing up/ sitting down for both parameters, nor in descending stairs for the MPF and walking for the amplitude (*p* > 0.05).

**Conclusion:**

The MPF pre-post differences of the exercise intervention were higher compared to the MPF pre-post differences of the placebo treatment. The amplitude pre-post differences were lower in the exercise intervention. In particular, the sound amplitude might be an indicator for therapy effects in persons with knee osteoarthritis.

**Trial registration:**

The study was retrospectively registered in the German Clinical Trials Register (DRKS00022936, date of registry: 26/08/2020).

## Introduction

Osteoarthritis of the knee is a complex, multifactorial disease characterized by a progressive degeneration of articular cartilage (including synovia and surrounding tissues). This degeneration is associated with higher kinetic friction of the joint. Persons suffering from knee osteoarthritis are often affected by chronic pain, stiffness, decreased quadriceps muscle strength, impaired postural control and joint proprioception leading to a progressive loss of function and difficulties in performing usual activities of daily life [1–3].

These symptoms can be treated through exercise. Therapeutic exercises have been shown to improve function (e.g. stiffness, restricted mobility) and to reduce knee pain with a low risk of negative side effects [4]. Neuromuscular training is able to acutely improve the sensorimotor control of the trunk and lower limbs and, thus, the quality of movement performance [5]. Beyond such short- and long-term effects on function and pain, long-term effects of exercise therapy on knee joint loading, assessed by changes in knee moments, may also be achievable [6].

Vibroarthrography reliably [7] measures vibrations and sounds in a synovial joint (diarthrosis) arising from the articulation of joint surfaces [8]. Hence, knee joint sounds reflect not only structural changes (e.g. roughness, degeneration), but also the function of the joint (e.g. lubrication) through intra- and extra-articular changes in friction. The amplitude of vibroacoustic signals steadily rises with the increasing degree of joint deterioration [9] and with the increasing level of knee load during standardized movements and activities of daily living [10, 11]. Moreover, vibroarthrography can mirror knee moments when the signal is controlled for several potential confounders (age, physical activity regarding knee loading, osteoarthritis stage) [12], which appears to be related to the joint’s contact stress and kinetic friction. On the contrary, decreases in compressive loads between articular surfaces and improvements in arthrokinematics are considered as friction-reducing properties of diarthrosis and may, therefore, result in decreased knee acoustic emissions [13].

So far, it is still unclear if therapeutic exercise affects knee acoustic emissions which are closely related to the arthrokinematics of the joint. More concretely, it is unknown if the acutely-induced exercise therapy effects in persons with knee osteoarthritis can be assessed by vibroarthrography. Therefore, the present research investigated the acute effect of a single bout of a neuromuscular exercise therapy on knee joint sounds during different activities of daily living. We hypothesize that the exercise therapy reduces knee acoustic emissions, in particular the amplitude, when compared to a placebo-controlled intervention and when relevant confounders are controlled.

## Methods

### Study design and ethics

The present study is a single-center, double-blinded, randomized controlled crossover trial aimed to investigate acute exercise effects on knee joint sounds. We implemented a non-invasive placebo control (laser needle acupuncture) to differentiate specific from non-specific treatment effects. The study was conducted according to the ethical guidelines of the Helsinki Declaration (with its modification up to Fortaleza, 2013) and was approved by a local ethics committee. Inclusion started in September 2019 and data collection ended in November 2019. All participants were familiarized with the study protocol and provided written informed consent. We registered this trial in a Clinical Trials Register (DRKS00022936, date of registry: 26/08/2020).

### Participants

Adults with confirmed unilateral or bilateral knee osteoarthritis were recruited via personal addressing. The diagnosed knee osteoarthritis stages were clinically screened beforehand and classified by a second investigator on the basis of the medical report and in accordance to the Kellgren-Lawrence classification. Included participants had a diagnosed knee osteoarthritis stage between one and four at the medial tibiofemoral and/or patellofemoral joint. Exclusion criteria encompassed fracture of the knee joint (e.g. tibial plateau, patella), knee injuries or surgery in the last 12 months, muscle soreness, diseases other than knee osteoarthritis that may affect the walking ability/standing stability, intense physical activity or medication modifying pain perception and proprioception in the last 48 h and acute inflammation of the knee joint.

### Experimental protocol

Each participant was scheduled for two visits at the same time of the day, with a wash-out phase of seven days in between the two visits. On one day, the participants received an exercise intervention treatment and on the other day a placebo treatment was applied. The order was randomized using a non-stratified full randomization order (BIAS for Windows, University of Frankfurt, Germany). Both treatments were performed in the same laboratory and lasted for 30 min.

For the exercise intervention, all participants performed six different neuromuscular exercises. For the placebo treatment, all participants were treated with a non-functional laser acupuncture device. During the placebo treatment, all participants completed self-report questionnaires about their physical activity level, disease-related characteristics and potential confounders. Before and after both, the placebo and intervention treatments, self-reported outcomes (questionnaires) and knee joint sounds were measured during three activities of daily living: five times standing up and sitting down (90° knee angle), level walking (6 m walkway) and going three steps downstairs (step depth: 27 cm, step height: 18 cm, step width: 120 cm; DIN standard for steps).

All three assessment activities were performed at a self-selected and comfortable speed. The leg (side) to be assessed was identical for every measurement and was randomly selected when the participant had osteoarthritis in both knees, otherwise the osteoarthritic knee was selected.

### Placebo treatment and blinding procedure

We used a placebo laser acupuncture as a control for the unspecific effects on knee joint sounds. Laser acupuncture is a popular method to relieve pain in different musculoskeletal diseases and has been shown to effectively reduce knee pain in persons with knee osteoarthritis in the short term [14]. It is, thus, likely that participants believe to receive a real treatment. All participants sat down on a physiotherapy bench while a laser acupuncture device was connected to their measured leg. Placebo laser acupuncture diodes were attached to three acupuncture points (the medial and lateral joint spaces and below the patella/patellar tendon) using a laser needle system (Laser ACUbeam, Laser Acumed GmbH, Beverungen, Germany). The device consists of three optical fibers that have a top section resembling a small needle. These laser diodes were fixed onto the skin at the above defined points with special adhesive tape. The acupuncture control device was turned on, but the needles were turned off and, therefore, emitted no laser. Participants and the acupuncturist had to wear protective glasses during the treatment and were told that the glasses kept them from seeing the emitted infrared laser light. Consequently, neither the participants nor the investigator knew of the placebo treatment (double blinded).

### Exercise intervention treatment

The neuromuscular exercises aim to improve the sensorimotor control of the trunk and lower limbs, as well as the quality of movement performance, while dynamically and functionally strengthening the lower limb muscles [15]. Participants learn to improve the motor control of their knee and hip joint controlling muscles by practicing a more neutral knee positioning (individual in view of lower limb alignments) during movement. Therefore, they were instructed to position their knee over the foot and to avoid a medial or lateral movement of the knee in relation to the foot [15].

The whole neuromuscular training consisted of six exercises, each with four different intensity levels (Table 1). Before starting the exercise intervention, the therapist determined the individual level of difficulty for each participant; exercise number five, as the most difficult exercise regarding knee alignment, was exemplarily performed and rated. Although some discomfort would be expected, the exercises should be performed within tolerable pain levels which should not exceed the participant's regular pain levels. If a specific exercise was found to aggravate the participant’s pain perception, the therapist reduced the level of the exercise. The entire exercise program was performed with both limbs and took 30 min to complete. The current exercise intervention was developed and described in more detail by Bennell et al. [5] and has been validated in studies with persons suffering from osteoarthritis [6, 15].

**Table 1:** Neuromuscular exercise intervention program. For a higher quality of movement, participants were constantly instructed to position their knee over the foot and to avoid a medial (valgus) or lateral (varus) position of the knee in relation to the foot (neutral knee positioning)

Exercise	Description	Sets, Frequency, Break between sets
1. Forward and backward sliding or stepping	Standing on the affected leg and sliding the opposite leg forward and backward (level 1) or stepping with the opposite leg forward and backward (level 3) while bending and straightening the affected side. Affected leg is nearly straight when feet are together and bent when feet are apart. Progression of level 1 (level 2) or level 3 (level 4) is achieved by adding an elastic resistance band around the affected leg to apply a varus-directed force during the movement. The participant is required to counteract in order to maintain the knee in the neutrally aligned position	3 sets of 10 reps, break of 30–60 s between sets
2. Sideways exercises	Standing on the affected leg and sliding or stepping the opposite leg forward and backward (level 1). Progression is achieved by adding an elastic resistance band around the affected leg to apply a varus-directed force during the movement. The participant is required to counteract in order to maintain the knee in the neutrally aligned position (level 2). Further progression is to additionally stand on foam (level 3) and closing the eyes during the movement (level 4)	3 sets of 10 reps, break of 30–60 s between sets
3. Functional hip muscle strengthening	Standing sideways to a wall with the non-affected leg closest to the wall so that the hip, thigh and knee are all slightly bent and touching the wall. Slightly bend affected knee (15–20°) and push leg into the wall and hold (isometric abduction, level 1). For progression, slowly bend and straighten affected knee while maintaining the push (level 2). For further progression, stepping sideways in both directions with a low resistance elastic band (level 3) or a higher resistance elastic band (level 4) around ankles, maintaining the slight knee bend	Level 1 + 2: 20 s hold, short break between efforts, two sets of 5 reps, break of 30–60 s between sets Level 3 + 4: total of 30 steps in each direction
4. Functional knee muscle strengthening	Standing with back to the wall, feet hip-width apart and one foot length away from the wall. Slowly sliding down the wall to about 30° knee bend, then sliding up again (level 1). For progression, shifting weight to be more over the affected leg (level 2). For further progression, rising from sitting down position on a chair (feet parallel, level 3) and with increased weight on the affected leg (feet in step position, level 4)	3 sets of 10 reps, break of 30–60 s between sets
5. Step-ups and touch-downs	Placing affected leg onto a step. Slowly stepping up onto the step. Touching opposite foot to the step then stepping back down slowly to the starting position (level 1). Stepping up on a higher step (level 2). For further progression, standing on the step and touching non-affected side to the floor in front and then behind the step (level 3). Touch-downs from a higher step (level 4)	3 sets of 10 reps, break of 30–60 s between sets
6. Balance	Lifting opposite side in front off the floor and balance on the affected leg (level 1) or stepping forward and lifting opposite side behind (level 2), with progressions adding arm movements (level 3) or stepping forward onto foam (level 4)	2 min practice

### Baseline self-report questionnaires

The German short form of the International Physical Activity Questionnaire (IPAQ-SF) records four intensity levels of physical activity in the last seven days: 1) vigorous-intensity activity, 2) moderate-intensity activity, 3) walking and 4) sitting. The sums of 1) and 2) were cumulated to calculate the amount of moderate to vigorous physical activity (MVPA) in minutes per week.

The Tegner Activity Score (TAS) contains a zero to ten point scale (0 = low level activity regarding knee loading, while 10 = highest possible level of activity regarding knee loading) to assess the knee demanding activity level from daily living to high level competitive sport [16]. The German version of the TAS has been shown to be reliable and valid [17].

Finally, the German version of the Western Ontario and McMaster Universities Arthritis Index (WOMAC, 24 items) is a reliable and valid questionnaire and was used to assess symptoms and physical functional disability caused by the participant's knee osteoarthritis [18].

### Self-reported outcomes

At both visits, acute knee pain was assessed before measurement, during pre-measurement, during intervention, during post-measurement and after measurement. The Visual Analogue Scale (VAS) is a reliable and valid single‐item measure of pain intensity which has been widely used in diverse adult populations, including persons suffering from osteoarthritis. The VAS consists of a 10 cm line with a starting point marked as “no pain” (score of 0 cm) and an endpoint marked as “worst imaginable pain” (score of 10 cm) [19].

Likewise, we recorded the participant's muscle fatigue after the exercise intervention by using a VAS consisting of a 10 cm line with a starting point marked as “no muscle fatigue” (score of 0 cm) and an endpoint marked as “worst imaginable muscle fatigue” (score of 10 cm).

### Vibroarthrographic recording

**Fig. 1:**
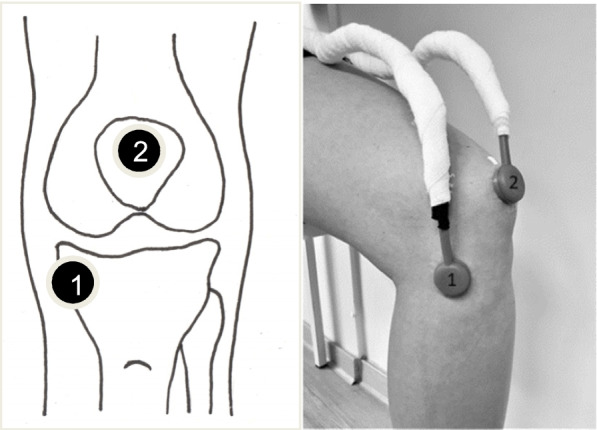
Microphone locations. **1** medial tibial plateau, **2** patella

Knee acoustic emissions were recorded using two high-performance, top port silicon acoustic sensors (Micro Electro Mechanical System (MEMS) microphones, size: 3 cm in diameter and 1 cm in depth, SPU0414HR5H-SB, Knowles Electronics, LLC. Itasca, IL, USA). Based on Kalo et al. [7], one microphone was placed on the medial tibial plateau and one in the center of the patella (Fig. 1). Both locations were palpated by the same experienced investigator for each participant and treatment and were controlled for equidistance by a second, experienced investigator. These specific locations showed an excellent intra-session reliability for both amplitude- and frequency-based outcomes (intraclass correlation coefficient (ICC)_2,1_: 0.73–0.95, standard error of measurement (SEM): 6–8%, coefficient of variation (CV): 0.02–0.07) [7]. The skin was prepared by hair removal (if needed) and cleansing with alcohol. Afterwards, the microphones were placed while the participant stood upright and attached to the skin using adhesive, double-sided tape (LSM2-ZKP-023, Löwenstein Medical Diagnostics GmbH, Germany). A flexible electrogoniometer (Penny and Giles Biometrics, Newport, UK) was used to quantify knee joint angles and knee motions.

### Data processing

The sound signals were digitized with a sampling rate of 16 kHz and transferred to a computer via an A/D converter (National Instruments Corp., Austin, TX, USA). The A/D converter was placed in a belt bag around the waist of the participant, with the wires attached to the thigh and knee. In order to avoid noise interference, the microphone wires were coated in noiseless fleece. This construction allowed natural movement during all activities.

For data processing, we used Matlab version R2018b (MathWorks, Natick, MA, USA). We extracted movement cycles by means of the knee angle to calculate a mean out of five repetitions of standing up and sitting down, the three middle stance phases out of all steps of level walking and the middle stance phase out of three steps downstairs, respectively. The signal was filtered using a digital band-pass filter with cut-off frequencies of 50 Hz and 1,000 Hz [12, 20]. The mean signal amplitude (dB) and the median power frequency (MPF, Hz) for each activity were calculated.

Both the amplitude and frequency signals were found to be highly reliable for intra-session values, but showed limited reproducibility for inter-day assessments [7]. We thus calculated intra-session (pre-post) differences for between-treatment comparisons. To determine pre-post-differences, the post-intervention values were substracted by the pre-intervention values.

### Statistical analyses

Our a priori sample size calculation (G*Power software version 3.1.9.2; Universität Düsseldorf; #11) was based on standard assumptions. We considered a two-sided alpha-error (α) level of 5%, a target effect size of 0.5, and an 80% power. Accordingly, we will need data from 19 participants (including an anticipated 10% drop out rate).

Before inference statistical analyses were commenced, data distribution and variance homogeneity were checked. According to Wellek et al. [21], we tested for carryover effects. To determine potential differences in the knee acoustic emissions between the placebo treatment and the exercise intervention treatment, we calculated analyses of covariance (ANCOVAs) using the pre-post-differences of all interval or pseudo-interval-scaled variables. Previous research with the same measurement setting has shown inconsistent interday reliability for the different sensor positions [7]. We, thus, used pre-post-differences (post substracting pre values) for further analyses. Based on Kalo et al. [12], we controlled for the participant's age, their knee demanding activity level (TAS) and their knee osteoarthritis stage at the medial compartment of the tibiofemoral joint and the patellofemoral joint.

All analyses were perfomed in SPSS version 25 (IBM Corporation, New York, NY, USA); an alpha-error of 5% was considered as a relevant cut-off significance value.

## Results

Twenty-eight (28) participants were screened. Five participants had to be excluded (surgery in the last 12 months or acute inflammation). From the included 23 participants, five withdrew informed consent without providing reasons.

The demographic characteristics and questionnaire data of the eighteen participants analyzed are shown in Table 2. Overall, 15 participants had a tibiofemoral knee osteoarthritis and 14 had a patellofemoral knee osteoarthritis. The knee osteoarthritis stages of all participants are summarized in Table 3. Three participants performed the neuromuscular exercise intervention on level 1, four participants performed the exercise on level 2, nine participants performed on level 3 and two participants performed on level 4.

**Table 2:** Participants’ characteristics

	**Mean**	**SD**	**Minimum**	**Maximum**
Age [years]	51.8	7.3	33	61
Sex	4 male, 14 female			
Weight [kg]	83.9	18.7	48.0	115.0
Height [cm]	172.8	9.0	160.0	196.0
BMI [kg/m^2^]	28.3	7.1	17.4	41.1
Duration of knee pain [years]	7.4	6.8	1	25.0
MVPA [min/week]	311	201	90	900
TAS [0–10]	3.9	0.7	3.0	5.0
WOMAC [0–240]	76.6	46.9	7.0	161.0
Muscle fatigue post treatment [0–10 cm scale]	3.8	2.3	0.4	8.4

*SD* Standard deviation, *BMI* Body mass index, *MVPA* Moderate to vigorous physical activity, *TAS* Tegner Activity Scale, *WOMAC* Western Ontario and McMaster Universities Arthritis Index

**Table 3:** Participants’ osteoarthritis stages

KOA stages	1	2	3	4
Tibiofemoral joint	0	1	4	10
Patellofemoral joint	1	6	5	2

Clinically diagnosed knee osteoarthritis (KOA) stage between one and four or in between two stages. Some participants had an osteoarthritis in both knee joints

We obtained no carryover effects between both interventions.

### Pain perception

**Fig. 2:**
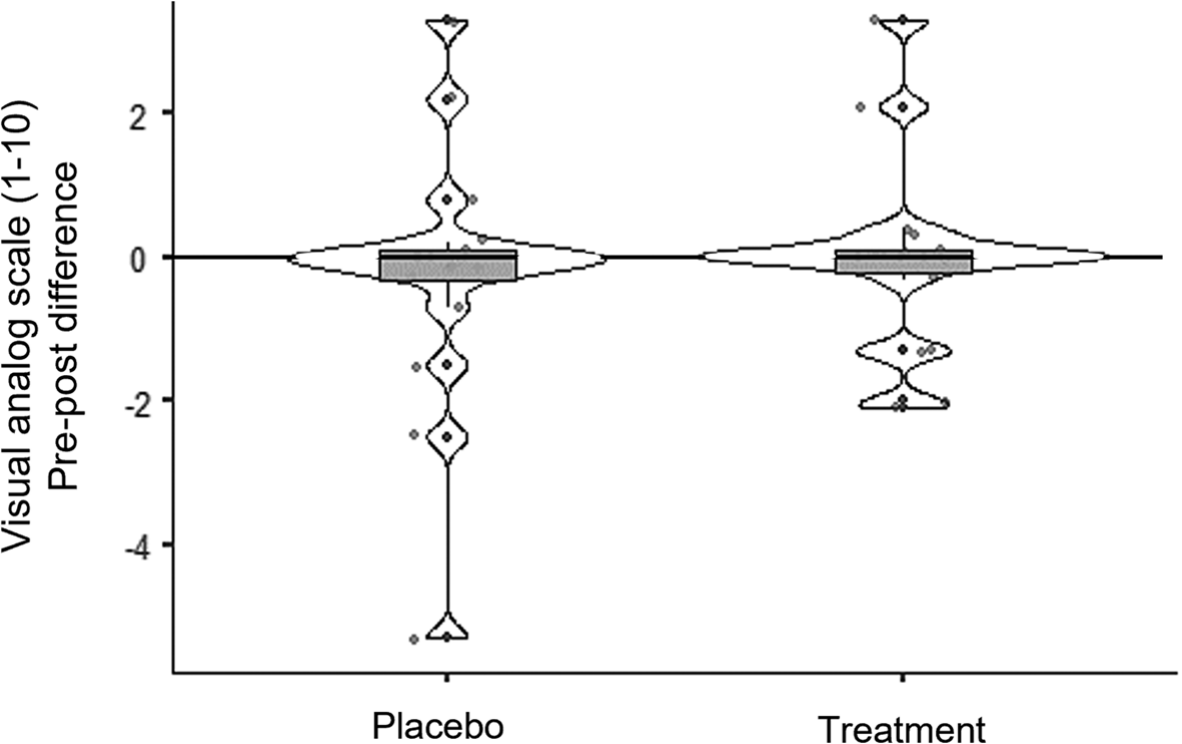
Pre-post differences of the pain intensity level**.** Comparison of placebo treatment and exercise intervention. Violin plots with median, interquartile ranges (boxes), individual values (dots) and data distribution

The mean pre-post-difference of pain intensity (± standard deviation) generated during the placebo treatment was -0.24 (± 1.82) points and during the exercise intervention -0.04 (± 1.27) points. The results showed no significant differences between the placebo treatment and the exercise intervention (*p* > 0.05). The pre-post-differences in pain perception during both interventions are shown in Fig. 2.

### Acoustic emissions at the medial tibial plateau

**Fig. 3:**
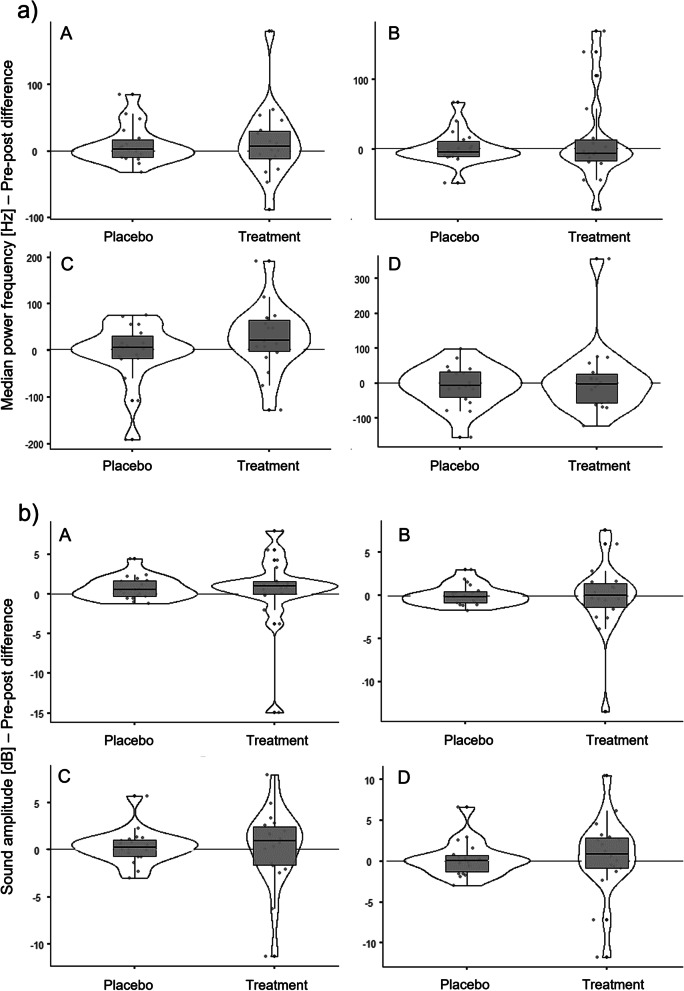
Pre-post differences of a) the median power frequency and b) the amplitude at the medial tibial plateau between placebo treatment and exercise intervention. Violin plots with median, interquartile ranges (boxes) and data distribution with individual values (dots). **A** = standing up, **B** = sitting down, **C** = walking, **D** = descending stairs

The mean (± standard deviation) pre-post-differences of the MPF generated during the placebo treatment were 9.55 (± 29.15) Hz (standing up), 2.78 (± 24.44) Hz (sitting down), -3.19 (± 65.08) Hz (walking) and -6.92 (± 61.30) Hz (stairs); during the exercise intervention the pre-post values were 13.01 (± 56.06) Hz (standing up), 13.65 (± 64.94) Hz (sitting down), 26.31 (± 71.09) Hz (walking) and 9.14 (± 101.82) Hz (stairs). In Fig. 3a) the pre-post-differences of the MPF at the medial tibial plateau of both treatments are depicted for comparison .

We obtained significant differences in the MPF pre-post-differences between the placebo treatment and the exercise intervention during standing up (F = 4.9, *p* < 0.05) when controlled for age and TAS and for sitting down (F = 4.9, *p* < 0.05) when controlled for TAS. There were no significant differences during walking (F = 0.1, *p* > 0.05) and descending stairs (F = 1.0, *p* > 0.05).

The mean (± standard deviation) pre-post-differences of the amplitude generated during the placebo treatment were 0.75 (± 1.43) dB (standing up), 0.07 (± 1.21) dB (sitting down), 0.35 (± 1.88) dB (walking) and 0.29 (± 2.20) dB (stairs); during the exercise intervention the pre-post values were 0.51 (± 4.68) dB (standing up), -0.16 (± 4.36) dB (sitting down), 0.23 (± 4.25) dB (walking) and 0.67 (± 4.83) dB (stairs). Fig. 3b) depicts the pre-post-differences of the amplitude at the medial tibial plateau of both treatments for comparison.

We obtained significant differences in the amplitude pre-post differences between the placebo treatment and the exercise intervention during standing up (F = 5.0, *p* < 0.05) and sitting down (F = 4.7, *p* < 0.05) when controlled for age, TAS and osteoarthritis stage. There were no significant differences during walking (F = 2.2, *p* > 0.05) and descending stairs (F = 3.1, *p* > 0.05).

### Acoustic emissions at the patella

**Fig. 4:**
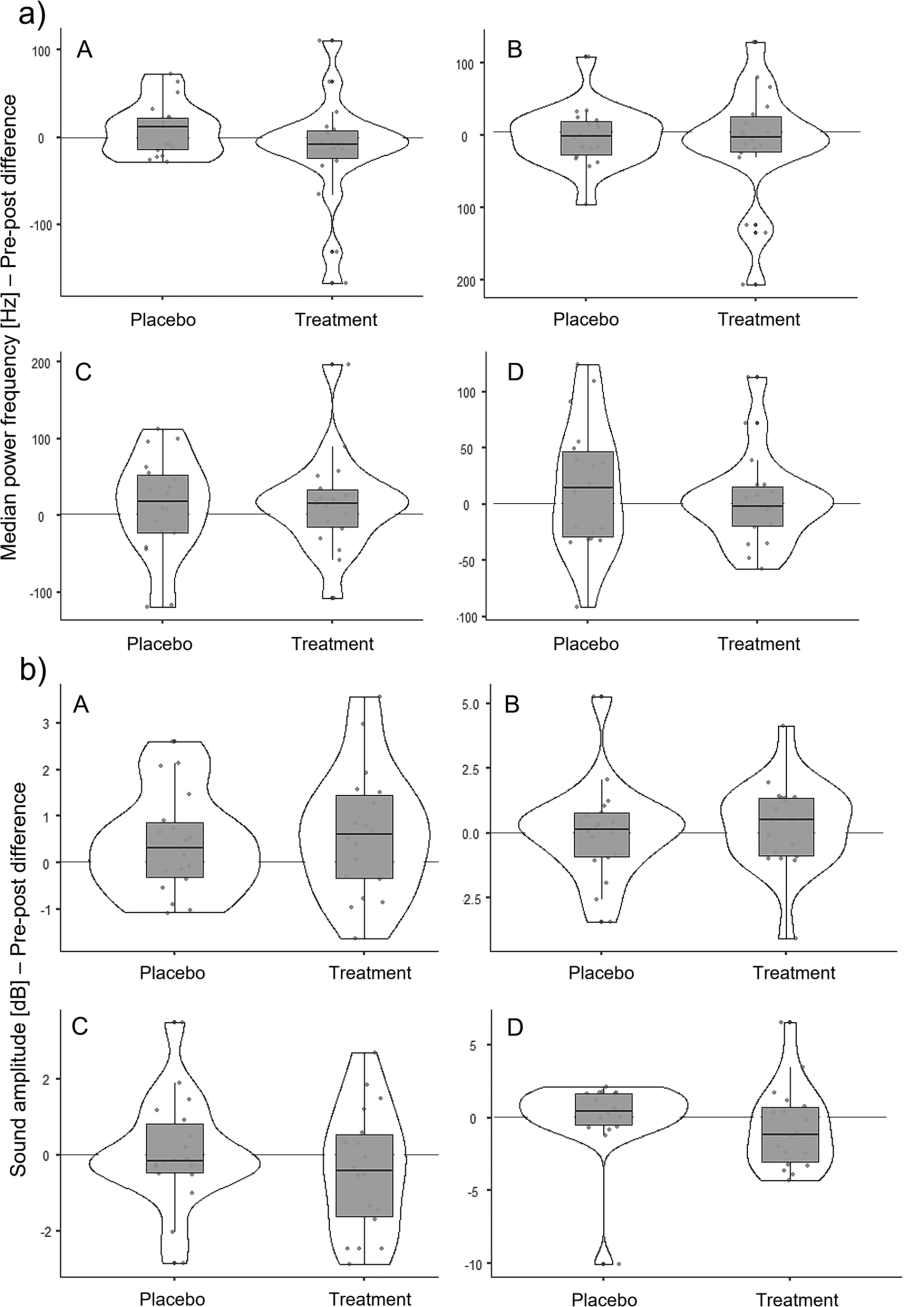
Pre-post differences of a) the median power frequency and b) the amplitude at the patella between placebo treatment and exercise intervention. Violin plots with median, interquartile ranges (boxes) and data distribution with individual values(dots). **A** = standing up, **B** = sitting down, **C** = walking, **D** = descending stairs

The mean (± standard deviation) pre-post-differences of the MPF generated during the placebo treatment were 9.82 (± 30.66) Hz (standing up), -1.62 (± 42.58) Hz (sitting down), 0.08 (± 1.42) Hz (walking) and 15.64 (± 57.11) Hz (stairs); during the exercise intervention the pre-post values were -13.82 (± 62.36) Hz (standing up), -11.59 (± 79.11) Hz (sitting down), 15.76 (± 64.25) Hz (walking) and 3.25 (± 41.19) Hz (stairs). Figure 4a) shows the pre-post-differences of the MPF at the patella of both treatments for comparison.

We obtained significant differences in the MPF pre-post differences between the placebo treatment and the exercise intervention during walking (F = 4.8, *p* < 0.05) when controlled for age. There were no significant differences during standing up (F = 0.1, *p* > 0.05), sitting down (F = 0.2, *p* > 0.05) and descending stairs (F = 0.1, *p* > 0.05).

The mean (± standard deviation) pre-post-differences of the amplitude generated during the placebo treatment were 0.41 (± 1.10) dB (standing up), 0.07 (± 1.89) dB (sitting down), 0.08 (± 1.42) dB (walking) and 0.02 (± 2.72) dB (stairs); during the exercise intervention the pre-post values were 0.63 (± 1.37) dB (standing up), 0.30 (± 1.73) dB (sitting down), -0.43 (± 1.64) dB (walking) and -0.73 (± 2.84) dB (stairs). Figure 4b) depicts the pre-post-differences of the amplitude at the patella of both treatments for comparison.

We obtained significant differences in the amplitude pre-post differences between the placebo treatment and the exercise intervention during descending stairs (F = 4.9, *p* < 0.05) when controlled for age and osteoarthritis stage. There were no significant differences during standing up (F = 0.2, *p* > 0.05), sitting down (F = 0.006, *p* > 0.05) and walking (F = 1.2, *p* > 0.05).

## Discussion

The purpose of this study was to examine the influence of an acute neuromuscular exercise therapy on knee joint sounds compared to a placebo control and during different activities of daily living. The neuromuscular exercise therapy showed some significant effects on knee acoustic emissions, while only a small part of our data set reached statistical significance.The participants’ perceived pain intensity changes from pre- to post-treatment were not different between the placebo and exercise intervention. In contrast, we found some significant differences in knee acoustic emissions between the placebo and the exercise intervention for both sensor locations when controlled for age, TAS and osteoarthritis stage. The MPF pre-post differences of the exercise intervention were higher compared to the MPF pre-post differences of the placebo treatment. Opposing results were observed for the amplitude pre-post differences, which were lower in the exercise intervention.These results are in line with our hypothesis. When considering the data distribution of the violin plots, the pre-post differences of the placebo control show a much more homogeneous response of individual knee joint sounds compared to the exercise intervention, especially at the medial tibiaplateau.

### Comparison with existing evidence

Performing a long-term neuromuscular exercise program has been shown to positively affect the knee pain and function of persons suffering from osteoarthritis. Our results revealed no differences in knee pain following a single bout of neuromuscular exercise intervention compared to a placebo treatment. This is in line with Goddard et al. [6], observing no change in knee pain after a single session of neuromuscular exercises. The absence of pain suggests that there are no contraindications to specific exercises for persons with knee osteoarthritis and, therefore, no pain-induced changes in movement quality.

In contrast to pain, a single bout of neuromuscular exercise has been shown to acutely improve the dynamic balance performance in individuals with unilateral knee OA [6]. The acute effects of exercise on knee acoustic emissions have, as yet, not been researched. Considering long-term-effects, one recent research has investigated the impact of an intermediate term, 2-week rehabilitation program on immobilization-related deterioration of the knee analyzed by vibroarthrography. The authors detected lower values of all measured sound parameters after the intervention (including amplitude and spectral power) [22]. Immobilized knees, when analyzed before rehabilitation, generated signals characterized by significantly higher values of VAG parameters (variability of the VAG signal, signal amplitude, frequency characteristics) in comparison to a control group. After the rehabilitation program, the participants with immobilized knees showed significant improvement in all VAG parameters compared to the baseline condition [22]. These findings are somewhat comparable to our results regarding the amplitude, but are in contrast to our frequency findings (which were generally increased by exercise compared to control in our sample). However, the authors had measured knee acoustic emissions only at the patellofemoral joint and, moreover, they had examined the spectral power at two distinct frequency bands while we had examined the MPF in a bandpass filtered data set between 50 and 1000 Hz. Compared with the investigation by Bączkowicz et al. [22], our participants performed just one bout of exercise training which may affect the lubrication and the arthrokinematic rather than obtaining long-term exercise effects such as muscle strengthening.

Altered neuromuscular function is thought to result in more variable contact loads at the articulating surfaces of the knee joint and, thus, elicit metabolic changes of the chondrocytes, compromising the mechanical properties of the cartilage [23]. Futhermore, neuromuscular exercise therapies may lead to a reduction in knee joint moments [15]. A decreased joint load has been found to result in a higher MPF and a lower signal amplitude [24]. These findings support our results in case of the amplitude and may imply that an acute neuromuscular exercise treatment leads to an improvement in movement quality and, therefore, decreased knee loading which might be quantifiable as reduced sound amplitude [10, 11]. In contrast, frequency-based parameters were shown to be able to discriminate between different types of activity [12, 25], but were also shown to be potentially independent of the knee load [10, 25].

### Physiological and exercise characteristics considerations

The exercise type, intensity and duration are all important factors in determining both the beneficial and harmful exercise effects on healthy and osteoarthritic joints [26, 27]. Considering the exercise duration, Mazor et al. [26] have indicated potential long-term changes in articular cartilage in response to exercise. Nevertheless, after only 30 min of neuromuscular exercise, we found a small change in knee acoustic emissions which potentially arose from decreased joint friction. When the intensity is focused, low and moderate, exercises appear to increase lubricin while high-intensity exercise decreases it [26]. We had a very heterogeneous collective and, hence, provided individualized exercise levels and, thus, intensities. Nevertheless, we should also take into account that, regarding muscle fatigue, the exercises were not demanding enough for some participants while being too exhausting for others. A wide range of perceived muscle fatigue was reported directly after the treatment intervention. Moreover, the data distribution of knee joint sounds showed a heterogeneous and individual reaction to the exercise intervention. The fatigue of muscles can lead to a decline in movement quality and, thus, to an increased knee loading. In addition, it should be considered that some participants may have benefited more from a lubrication effect than others.

### Practical implications

Vibroarthrography provides information on the functional state of diarthrosis and its articular cartilage under motion [28]. Taking into consideration that louder knee joint sounds reflect greater joint degeneration [9], an increased knee joint loading [10, 11] and/or a reduced movement quality [13], knee acoustic emissions could be clinically useful to assess treatment effects in knee osteoarthritis. Personalized exercise therapies may improve the arthrokinematic motion quality (mechanical factors) and cartilage metabolism (e.g. lubrication, anabolic activity) and, thus, slow down or even stop the progression of knee osteoarthritis. Moreover, an individual appropriate exercise intensity might be possible. Assessing acoustic emissions are not meant to replace common assessments, such as pain intensity or self-perceived function, but to complement them. This assumption is somewhat supported by our findings of the unchanged pain score (no between-group-differences) in contrast to the changes we found in the vibroarthrographic signal. The information provided by joint sounds allow a more detailed insight of knee joints, but these information is not just a surrogate of the pain intensity; one cannot be replaced by the other.

The MPF seems not to be influenced by the actual knee load [10], nor the cartilage status [25], but by the type of activity. Therefore, it seems challenging to use the MPF as an outcome parameter on acute treatment effects. In contrast, the sound amplitude has been shown to be an adequate indicator for knee loading as well as the cartilage status. Moreover, our results indicate an increase in MPF and a decrease in amplitude caused by exercise therapy. However, at the present state of knowledge, it is mandatory to better understand the origin of acoustic emissions and how they are affected by exercise.

### Limitations and future research

Only a small part of our data set reached statistical significance. Some reasons might be the individual exertion of our participants (i.e. lubrication vs. muscle fatigue), the duration of our exercise treatment and/or technical reasons like sensor attachment, hardware, signal processing.

Some studies detect knee acoustic emissions in osteoarthritis patients at frequencies between 100 Hz—10 kHz when using piezoelectric sensors [29]. Although our results were comparable to those of other studies [25], it is possible that measuring with higher sampling rates could lead to additional results.

Previous studies have recommended to control the movement speed and/or angular velocity for all participants because these may have an impact on knee acoustic emissions [12, 30]. Our participants chose a self-selected comfortable speed for all movements, therefore everyone could perform a controlled movement adjusted to their own knee pain, stiffness and function. Consequently, the movement speed was controlled within but not between participants and was, thus, not set on an objective basis but rather on a self-perceived one. Therefore, future studies may standardize the movement speed/angular velocity [12]. Since the lubricant properties of synovial fluid prevent a temperature increase due to the friction of articulating joint surfaces, a change in temperature of the knee might indicate the lubrication status of the joint [31]. Future studies could measure the knee temperature before and after an exercise intervention to gain further insight into individual exercise responses. Teague et al. [32] have already validated a smart, multimodal knee brace for measuring knee health including acoustic signals, knee swelling and skin temperature.

A smaller data distribution as a reaction of the neuromuscular exercise therapy and, therefore, more precise results could be created by a more homogeneous collective. Our main findings should be confirmed and further explored by future studies additionally investigating the long-term effects of neuromuscular exercise therapies on knee acoustic emissions in persons suffering from knee osteoarthritis.

## Conclusion

The present results showed some significant differences of a neuromuscular exercise intervention on knee acoustic emissions compared to a placebo laser acupuncture, when controlled for age, TAS and osteoarthritis stage. The MPF differences tended to be higher in the exercise intervention when compared to the placebo treatment. The amplitude showed opposing results where pre-post-differences tended to be lower in the exercise intervention. In particular, the sound amplitude might be an indicator for therapy effects in persons with osteoarthritis. Further studies are needed to confirm our results on the effect of acute exercise therapy on knee acoustic emissions; these studies should standardize the measurement process particularly movement speed/angular velocity, measure muscle activity (including muscle fatigue), knee temperature and investigate the effects of a long-term exercise treatment.

## Data Availability

The datasets generated and/or analyzed during the current study are not publicly available but are available from the corresponding author on reasonable request.

## Abbreviations

BMI: Body mass index
IPAQ-SF: Short form of the International Physical Activity Questionnaire
MPF: Median power frequency
MVPA: Moderate to vigorous physical activity
OA: Osteoarthritis
SD: Standard deviation
TAS: Tegner Activity Score
VAS: Visual Analogue Scale
VAG: Vibroarthrography
WOMAC: Western Ontario and McMaster Universities Arthritis Index
